# A novel *adipose* loss-of-function mutant in *Drosophila*

**DOI:** 10.1080/19336934.2024.2352938

**Published:** 2024-05-13

**Authors:** Nicole A. Losurdo, Adriana Bibo, Jacob Bedke, Nichole Link

**Affiliations:** Department of Neurobiology, University of Utah, Salt Lake, UT, USA

**Keywords:** *Drosophila*, *adipose*, neurodevelopment, fat body, lipogenesis, CRISPR

## Abstract

To identify genes required for brain growth, we took an RNAi knockdown reverse genetic approach in *Drosophila*. One potential candidate isolated from this effort is the anti-lipogenic gene *adipose* (*adp*). Adp has an established role in the negative regulation of lipogenesis in the fat body of the fly and adipose tissue in mammals. While fat is key to proper development in general, *adp* has not been investigated during brain development. Here, we found that RNAi knockdown of *adp* in neuronal stem cells and neurons results in reduced brain lobe volume and sought to replicate this with a mutant fly. We generated a novel *adp* mutant that acts as a loss-of-function mutant based on buoyancy assay results. We found that despite a change in fat content in the body overall and a decrease in the number of larger (>5 µm) brain lipid droplets, there was no change in the brain lobe volume of mutant larvae. Overall, our work describes a novel *adp* mutant that can functionally replace the long-standing *adp*^*60*^ mutant and shows that the *adp* gene has no obvious involvement in brain growth.

## Introduction

Many genes associated with human disease were discovered and studied through model organisms, including genes required for brain growth [1–4]. Human variants in critical neurodevelopmental genes can cause microcephaly, which is a rare neurodevelopmental disorder characterized by a reduced occipital frontal circumference (OFC) of two standard deviations (SD) or more below the mean for a child’s age and sex [5–7]. Half of all known causes of microcephaly are from genetic mutations, suggesting that additional cases of genetic microcephaly might be found by assessing genes for neurodevelopment defects in model organisms [3,4,6–8]. We hypothesized that by using a reverse genetics approach, we would isolate novel genes required for neurodevelopment and linked to human disease. We used commercially available *Drosophila* RNAi lines to knock down candidate genes in developing brains and screened third-instar larval *Drosophila* for brain size differences. Through this screen, we identified a potentially novel candidate gene in neurodevelopment, *adipose* (*adp*).

*Adipose* was first characterized in the 1950s when a wild strain of fly, *adp*^*60*^, was isolated in Africa [9]. These *adp*^*60*^ flies had a 23-base pair deletion in the middle of the *adp* gene, leading to increased lipogenesis [10–12]. Adult *adp*^*60*^ flies were resistant to starvation compared to Oregon R controls, and both the larvae and adults were shown to have higher triglyceride levels [10–12]. Further work validated that *adp* functions as an inhibitor of lipogenesis, and the *adp*^*60*^ flies carried a loss of function allele, demonstrating an increase in fat storage. While the lipogenesis phenotypes of *adp*^*60*^ have been well characterized, it is currently unknown whether *adp* plays a role in neurodevelopment.

The importance of fat during brain development has been well demonstrated across species. In vertebrates, fat is necessary to produce the myelin sheath covering axons. In the developing fly brain, neural stem cells, called neuroblasts, reside in stem cell niches protected by lipid droplets [13,14]. No work has shown whether *adp* is necessary to produce brain lipid droplets in larval *Drosophila*, but we hypothesized that *adp* is necessary for neurodevelopment and is likely to function through lipid droplet production in the brain. In this paper, we generated a novel *adp* mutant that behaves as a loss of function mutant that replicates high-fat content as previously shown. We also demonstrate that *adp* is not necessary for neurodevelopment in the fly nor for lipid droplet production in the brain.

## Results

**Figure 1.**
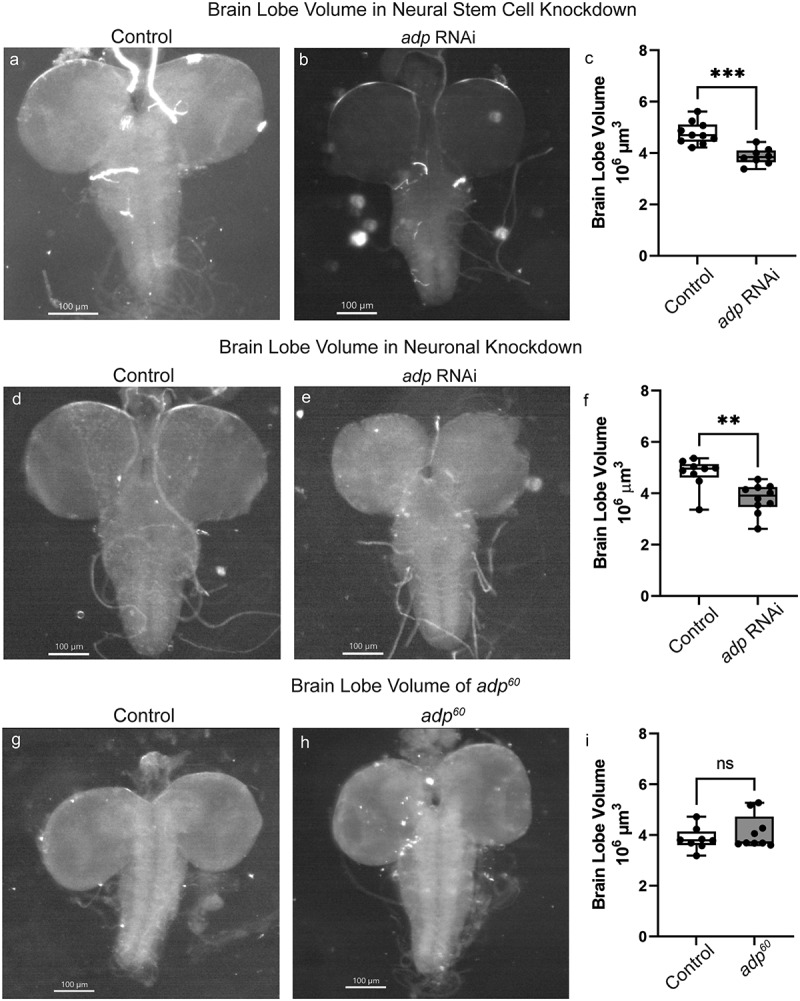
RNAi knockdown of *adp* but not the presumed *adp*^*60*^ mutant results in significantly reduced brain lobe volume. Stereroscope images of third-instar larval brains just prior to pupation with (A, D) GFP knockdown (VALIUM22-EGFP.ShRNA.1) and (B, E) *adp* knockdown (TRiP.HMC006600) in neural stem cells (A-C, *insc-GAL4*) or post-mitotic neurons (D-F, *nSyb-GAL4*). (C) Brain lobe volume of neural stem cell knockdown of GFP (control) or *adp* in third-instar larvae; each dot represents one brain lobe (*n* = 8–10). Knockdown of *adp* in neural stem cells results in significantly reduced brain lobe volume compared to control (independent t-test, *t* = 4.932, df = 16, *p* = 0.0002). (F) Brain lobe volume of post-mitotic neuronal knockdown of GFP (control) or *adp* in third-instar larvae, each dot represents one brain lobe (*n* = 9–10). Knockdown of *adp* in post-mitotic neurons results in significantly reduced brain lobe volume compared to control (independent t-test, *t* = 3.694, df = 17, *p* = 0.0018). Stereoscope images of third-instar larval brains just prior to pupation of (G) *Oregon-R* and (H) the presumed *adp*^60^ fly stocks. (I) Brain lobe volume of *Oregon-R* (control) or *adp*^*60*^ third-instar larvae, each dot represents one brain lobe (*n* = 8–9). The presumed *adp*^*60*^ mutant does not differ in brain lobe volume compared to an *Oregon-R* control (independent t-test, *t* = 0.9658, df = 15, *p* = 0.3495). Sequencing of the presumed *adp*^*60*^ stock showed no mutation in the *adp* gene.

In an effort to identify conserved pathways required for brain growth and novel players in neurodevelopment, we screened a collection of *Drosophila* genes for function in the brain using *in vivo* RNAi. We crossed *UAS-RNAi* flies to either *inscuteable-GAL4* (*insc-GAL4*) [15] or *neuronal Synaptobrevin-GAL4* (*nSyb-GAL4*) to knock down genes of interest in neuronal stem cells or post-mitotic neurons, respectively. Developing brains from late third-instar larvae were collected for volumetric analysis to assess brain lobe growth [8,16]. Knockdown of *adipose* (*adp*) in both neuronal stem cells and post-mitotic neurons resulted in significantly reduced brain lobe volume compared to the control knockdown of GFP (Figure 1a–f).

Generally, total larval number in each vial is not controlled in our standard RNAi crosses. However, competition for resources can influence animal and tissue growth in some mutants and conditions [17]. To test whether *adp* RNAi brain volume was affected by growth conditions, we determined how larval crowding modified our results. Since neuronal *adp* knockdown had the most robust results, we looked at brain size in a medium animal density (50–65 embryos) and high animal density (150 embryos) condition and found that control (GFP RNAi) brain volume was not different between crowding conditions (Supplemental Figure S1a), indicating that brain volume in general is not affected by crowding. However, *adp* knockdown animals had reduced brain volume that was significantly smaller than control in the high animal density condition (Supplemental Figure S1c), but not the medium animal density condition (Supplemental Figure S1b). Therefore, Adp regulation of brain growth is affected by crowding and competition for resources. Together, these results indicate that *adp* is involved in neurodevelopment.

To support the idea that Adp is important during neurodevelopment, we determined which cell types express *adp* in the larval brain. We generated 36 single molecule fluorescent *in situ* hybridization (smFISH) DNA primary probes spanning the *adp* mRNA region and a single fluorescently labelled secondary probe to use in larval brain RNA *in situ*. *adp* RNA appears to be expressed throughout the brain at a moderate level with no cell specificity (Supplemental Figure S2a,b). To ensure the documented signal was due to the presence of RNA and was not background, we treated larval brains with RNase prior to smFISH and found the signal was almost completely abolished (Supplemental Figure S2c). These results indicate the smFISH signal in (Supplemental Figure S2a,b) is RNA and likely the *adp* transcript. Interestingly, our *adp* smFISH probes also produce bright puncta in the nucleus of some cells which are not eliminated after treatment with RNase. These puncta probably correspond to the *adp* DNA locus and serve as an ideal control for hybridization.

To verify that reduced brain lobe volume resulted from a loss of *adp* and not off-target RNAi effects, we assessed a known loss-of-function mutation called *adp*^*60*^ [9,10]. Adult male *adp*^*60*^ flies are resistant to starvation, and both adult males and third-instar larvae have increased triglyceride content compared to wild-type animals, indicating that Adp functions to inhibit lipogenesis [10–12]. However, third-instar larval *adp*^*60*^ brains were not significantly different in size compared to *Oregon-R* control brains (Figure 1(g–i)). This negative result could mean that *adp* is either unnecessary for brain development or the mutation in *adp*^*60*^ may no longer be present.

We aimed to verify the mutation by sequencing the presumed *adp*^*60*^ fly with the same primers described in the initial characterization [11]. The published mutant contains a 23-base pair deletion that removes nucleotides 1153 to 1176 in exon 2, resulting in an early stop codon in the predicted protein. Sanger sequencing established that the *adp* genomic sequence in the available *adp*^*60*^ mutants matches the reference genome with no deletion present in both the main and backup *adp*^*60*^ Bloomington stocks, indicating that the presumed *adp*^*60*^ stock is no longer a mutant allele of *adp*.

**Figure 2.**
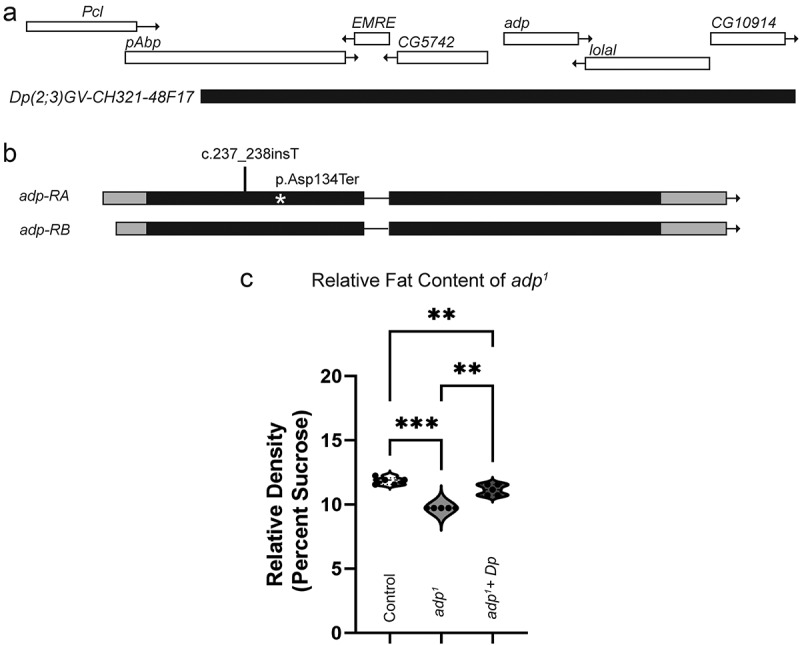
*adp*^*1*^ has higher fat content than controls. (a) Genomic region of *adp* and location of duplication line used for rescue. (b) Sequencing of fly *adp*^*1*^ showed a frameshift mutation (c.237_238insT) resulting in an early stop codon p.Asp134Ter marked with the asterisk. (c) Buoyancy assay comparing control (*w*^*1118*^), *adp*^*1*^, and *adp*^*1*^ plus the genomic duplication noted in (A), each dot represents a replicate experiment of around 20–30 larvae per genotype (total n sizes from all replicates: control *n* = 100, mutant *n* = 139, rescue *n* = 125). Loss of function *adp*^*1*^ larvae float at a lower density than control larvae, while a genomic duplication of fly *adp* is able to rescue (repeated measures one-way ANOVA, F = 117.9, dF = 14, *p* = 0.0001, with Tukey’s multiple comparisons control vs. *adp*^*1*^ *p* = 0.0002, control vs *adp*^*1*^ plus duplication *p* = 0.0033, and *adp*^*1*^ vs *adp*^*1*^ plus duplication *p* = 0.0035).

Due to the lack of an available *adp* mutant, we sought to generate our own loss-of-function mutant using the TRiP-CRISPR toolbox [18–28]. We crossed flies expressing Cas9 in the germline (*nanos-Cas9*) together with flies expressing single guide RNA for *adp* targeting base pairs 221–243 in the first exon. F1 males are expected to have germline mutations in *adp*, and F2 founder flies were isolated to generate 10 independent mutant *adp* lines. Initially, three lines were genetically characterized using PCR and Sanger sequencing of the *adp* locus. Surprisingly, all three contained small INDELs in the guide RNA target region, resulting in early stop codons in the predicted protein. *adp* INDEL lines were crossed to *w*^*1118*^ for three generations to remove potential off-target mutations. The remaining 7 lines have not been sequenced. We decided to focus on a single mutant, which we named *adp*^*1*^. The *adp*^*1*^ mutant contains a frameshift mutation (c.237_238insT) predicted to result in an early stop codon in the first exon (p.Asp134Ter) (Figure 2b). The *adp*^*1*^ mutant is homozygous fertile and viable. Due to how early the predicted truncation mutation appears, we predict *adp*^*1*^ to be a loss-of-function mutation.

Previous research shows that the loss of *adp* results in increased fat stores, so we verified this observation with our new mutation before assessing for neurodevelopmental phenotypes [9–12]. Using a buoyancy assay, we evaluated changes in fat storage at the third-instar larval stage [29]. *adp*^*1*^ mutant and *w*^*1118*^ control third-instar larvae were floated in a sucrose solution where density was increased until all larvae were floating. The density at which all larvae floated was recorded and analysed. As expected, *adp*^*1*^ mutants float at a lower density than *w*^*1118*^ controls, indicating an increase in fat content compared to controls (Figure 2c). To show that the increase in fat storage was due to mutations in *adp* and not background variability or off-target effects of CRISPR mutagenesis, we introduced a 80 kb genomic duplication line containing the *adp* locus and again tested buoyancy. We were able to significantly rescue the fat phenotype, indicating that the loss of *adp* causes increased fat stores (Figure 2c).

**Figure 3.**
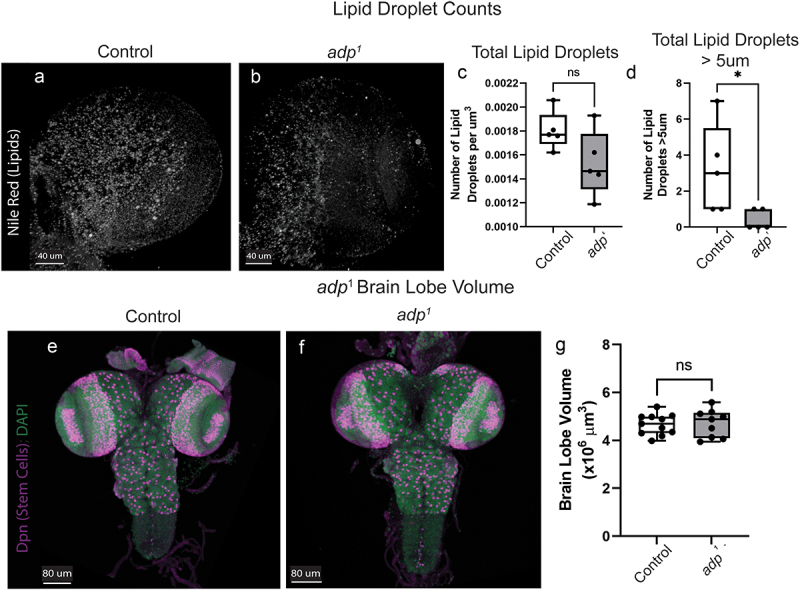
*adp*^*1*^ shows no difference in number of brain lipid droplets nor brain lobe volume. Nile red staining in the third-instar brain lobe showed no difference between (A) controls (*w*^*1118*^) and (B) *adp*^*1*^ in the total number of lipid droplets, quantified in (C), where each dot represents a single brain lobe (independent t-test, *t* = 1.154, dF = 8, *p* = 0.2819). (D) *adp*^*1*^ mutants have significantly less lipid droplets greater than 5 µm in diameter, where each dot represents a single brain lobe (independent t-test, *t* = 2.456, dF = 8, *p* = 0.0396). Immunohistochemistry staining of third-instar larval (E) *w*^*1118*^ control and (F) *adp*^*1*^ brains showing Deadpan (Dpn, stem cells, magenta) and DAPI (DNA, green). (G) Brain lobe volume of *w*^*1118*^ (control) or *adp*^*1*^ third-instar larvae, each dot represents one brain lobe (*n* = 9–11). The *adp*^*1*^ mutant does not differ in brain lobe volume compared to a *w*^*1118*^ control (independent t-test, *t* = 0.2020, df = 18, *p* = 0.8422).

Having confirmed that *adp*^*1*^ displays similar loss of function phenotypes based on previous research, we wanted to know if *adp* loss results in brain growth perturbations to validate our RNAi data. The larval brain contains lipid droplets that help maintain the stem cell niche, so we first assessed whether *adp*^*1*^ has changes in lipid droplet number that would indicate changes in lipogenesis in the brain [13,14]. We performed Nile Red staining in third-instar larval brains and quantified the total number of lipid droplets in *adp*^*1*^ and *w*^*1118*^ brains [13,30]. While *adp*^*1*^ mutants display no significant difference in the total number of lipid droplets per micrometre cubed, they do have significantly less droplets over 5 µm in diameter (Figure 3a–d). This suggests that there is a change in lipogenesis in the brain, and *adp* may be involved in controlling lipid droplet size

Finally, we wanted to determine if *adp* loss-of-function affected brain growth to replicate our RNAi data in a loss-of-function model (see Reagent Table for specific antibodies used). We quantified the brain lobe volume of *adp*^*1*^ and *w*^*1118*^ third-instar larvae but found no significant difference, suggesting that *adp* is not necessary for brain growth (Figure 3e–g). Interestingly, *adp*^*1*^ animals are generally healthy and survive well at a high animal density, suggesting that the crowding effect documented in *adp* knockdown (Supplemental Figure S1) does not replicate in *adp* mutant animals. Our results also suggest that the initial RNAi results we found may have been due to off-target effects or a result of cell-specific knockdown.

## Discussion

In this study, we generated a novel *adp* mutant to investigate its role in neurodevelopment. We showed that the previous standard *adp* mutant available from the Bloomington Stock centre, *adp*^*60*^, did not carry the described mutation. Our new mutant, *adp*^*1*^, acts as a loss-of-function exhibiting the expected phenotype of increased fat stores but failed to show any changes in neurodevelopment.

Previous discovery and description of the *adp*^*60*^ fly demonstrated *adp’s* role in the negative regulation of lipogenesis [9–12]. Adult *adp*^*60*^ flies had increased survival in starvation scenarios, and both larvae and adults exhibited increased triglyceride storage in the fat body. This research validated the use of *adp*^*60*^ as a negative control for lipogenesis research [29]. When we looked at brain lobe volume of *adp*^*60*^ compared to *Oregon R* controls, we found no difference. We do note that the *Oregon R* controls are significantly smaller than other controls used in this study. However, we found no difference in *adp*^*60*^ brain lobe volume compared to all other controls in this study. We hypothesize that brain lobe volume difference in *Oregon R* flies could be due to background variation. We do not believe background variation or off-target mutations affect *adp^1^* [1 because it was backcrossed to *w*^*1118*^ for three generations. Since we did not see a reduction in brain lobe volume in the presumed *adp*^*60*^ line, we wanted to validate the presence of the characterized *adp*^*60*^ mutation. Our sequencing of the *adp*^*60*^ stock obtained from the Bloomington Stock Center using the primers described in the original publication showed sequence that was identical to the reference genome [11]. The 23 base pair deletion was no longer present in this stock, making it wild-type. In order to perform our own tests of the role of *adp* in neurodevelopment, we generated a new loss-of-function mutant.

Our *adp*^*1*^ mutant larvae float at a lower density than controls, equating to a higher fat-to-muscle ratio, consistent with an *adp* loss-of-function phenotype (Figure 2C). Despite the previous literature on *adp*^*60*^ not specifically quantifying the buoyancy of *adp* mutant larvae, most buoyancy protocols suggest using *adp*^*60*^ larvae as a control for higher fat content [29]. These results validated that our newly generated mutant acts as a loss-of-function and demonstrated that our mutant could functionally replace *adp*^*60*^ as a control in lipogenesis research.

While *adp* has not previously been linked with neurodevelopment, expression data show that transcript and protein are present in the third-instar larval central nervous system [31–34]. This expression profile and our RNAi knockdown results (Figure 1a–f) indicated that *adp* might have a role in brain development. Since Adp is involved in lipogenesis, we assessed whether this change in brain size was partially due to crowded growing conditions. When looking at GFP knockdown alone, we saw no difference in brain size between medium and high animal densities, indicating that brain size in general is not affected by crowding (Supplemental Figure S1a). However, we did find that *adp* knockdown brain volume phenotypes are only present in high animal density conditions (Supplemental Figure S1c). Interestingly, even though *adp* mutants thrive in higher density conditions, no brain size difference can be documented. There may be some interplay with *adp* dosage, cell-specific knockdown, and larval crowding that affects the growth of the brain.

Lipid droplets provide protection for neuroblasts in hypoxic environments, allowing them to remain proliferative, and protect the neuroblasts from assault by reactive oxygen species [13,14]. Disruption of lipid droplet production in glia leaves neuroblasts vulnerable to hypoxic conditions [13]. Adp inhibits triglyceride storage but has not previously been linked to lipid droplet production in the larval brain. Here, we show that *adp* loss-of-function does not affect the total number of lipid droplets in the third-instar larval brain but decreases the number of droplets greater than 5 µm in diameter (Figure 3a–d), indicating that *adp* may regulate lipid droplet size.

Despite our RNAi data indicating that *adp* may function in the developing brain, we failed to see a difference in brain size in our mutant (Figure 3e–g). *Adp*^*1*^ acts as a loss-of-function mutant based on its mutation, fat phenotypes, and ability to rescue with duplication, so the lack of brain size phenotype confirms *adp* is not necessary for proper brain development. RNAi can have off-target effects, but genetic compensation can also occur. It has been shown in numerous organisms that phenotypic differences can exist between knockout and knockdown animals [35,36]. Compensatory genetic networks can arise in germline mutations allowing adaptation as the animal develops, which often negates deleterious effects [35,36]. Alternatively, post-transcriptional or post-translational effects might also prevent detrimental phenotypes [35,36]. Adp could also have different roles in different cell types in the brain. When Adp function is lost in the whole animal, some cell-type specific effects could balance out, leading to no obvious phenotypes in the mutant. Despite this, we are confident that *adp* is not a vital component of brain volume regulation.

## Methods

### Fly lines

The following fly lines were used: *adp* RNAi (*P{TRiP.HMC06600}attP40*), EGFP RNAi (*P{VALIUM22-EGFP.shRNA.1}attP40*), *inscuteable-GAL4* (*P{w[+mW.hs]=GawB}insc[Mz1407])* [15], *neuronal Synaptobrevin-GAL4* (*P{y[+t7.7] w[+mC]=nSyb-GAL4.P}attP2*), *adp*^*60*^ [9,10], *Oregon-R* [9,10], *nanos-Cas9* (*P{y[+t7.7] v[+t1.8]=nos-Cas9.R}attP2*) [18], *adp* snRNA:U6:96Ac (*P{y[+t7.7] v[+t1.8]=TKO.GS04840}attP40*) [18–28], *adp*^*1*^(this study), *w*^*1118*^ [37,38], *w[1118]; Dp(2;3)GV-CH321-48F17, PBac{y[+mDint2] w[+mC]=GV-CH321-48F17}VK00031*. All flies were maintained at 25°C and grown on Archon glucose formula medium in plastic vials. Crosses were performed at the temperature indicated (18°C, 25°C, or 29°C). Brain volume measurements were conducted in late wandering 3rd-instar larvae identified by gut clearance and extruding spiracles [8,16].

Due to the Bloomington *adp*^*60*^ stock no longer containing the described mutation, the authors asked multiple labs that previously worked with the allele for a copy, but none were able to provide one.

### RNAi knockdown of *adp*

Male *adp* RNAi and EGFP RNAi flies were crossed with either *insc-GAL4* or *nSyb-GAL4* females for knockdown in neural stem cells or post-mitotic neurons, respectively. Crosses were set at 29°C at the same time for each experiment. Third-instar larvae were selected for brain lobe volume analysis.

### Immunohistochemistry for brain volume

Late third-instar larval brains were dissected and immediately fixed in 4% paraformaldehyde in Phosphate Buffered Saline + 0.3% TritonX (PBST) for 20 min [8,16]. Brains were washed with PBST three times for 5 min and blocked twice with PBST + 1% w/v Bovine Serum Albumin (PBSTB) for 30 min before blocking with PBSTB + 5% Normal Donkey Serum (NDS) for 30 min. Brains were incubated with 1:1000 rat anti-Deadpan (neuroblast marker, Abcam, ab195173) in PBSTB overnight at 4°C. Primary antibody was removed from brains before washing three times with PBSTB for 20 minutes. Next, the brains are incubated with secondary antibodies 1:500 Donkey anti-Rat fluorophore 647 (Jackson ImmunoResearch Laboratories Inc., 712-605-153) and 1:1000 DAPI for one hour at room temperature. Finally, brains were washed with PBST four times for 10 min before being mounted for confocal microscopy.

#### Power analyses for brain lobe volume

We used the program G*Power to assess the power of our n sizes used in this study. We decided to use the data from the *nsyb-GAL4* knockdown to assess the power. We selected t tests for ‘Test Family’ and ‘Means: Difference between two independent means (two groups)’. We performed a ‘Post hoc: Compute achieved power – given α, sample size, and effect size’ for the ‘Type of power analysis’ since we are using previously collected data. We selected Two tails, and left the ‘α error probability’ as .05. Our n sizes were 9 and 10 for GFP RNAi and *adp* RNAi, respectively. We then calculated the Cohen’s d with their calculator. We typed in the averages (GFP RNAi = 4.791, *adp* RNAi = 3.803) and standard deviations (GFP RNAi = 0.5923, *adp* RNAi = 0.5730) and the software calculated d = 1.7006163. We then calculated the power which was 93.64%. We therefore conclude that our n sizes of around 10 brains per condition is sufficiently powered to detect differences in brain lobe volume.

### Larval crowding assay

Virgin female *nSyb-GAL4* flies were crossed with male *adp* RNAi or *EGFP* RNAi on grape plates set in embryo collection chambers at 29°C. Embryos were collected off grape plates 18–24 h later, prior to hatching [17]. Embryos were placed in blue food vials in two conditions: medium animal density (50–65 embryos) or high animal density (150+ embryos) [17]. The high animal density suggests crowding and competition for resources. Brains from late wandering third-instar larvae were isolated and measured for brain lobe volume as previously described.

### Generation of CRISPR mutants

The *adp*^*1*^ mutant was generated from the TRiP-CRISPR stocks and TRiP-CRISPR Knockout (TRiP-KO) protocol [18–28]. Ten *nanos-Cas9* females were crossed with 6 *adp* sgRNA males at 25°C to generate germline mutations in *adp* [18–21,24,27]. Fifteen F1 male flies (*y,v,sc,sev; adp sgRNA/+; nanos-Cas9/+*) were then crossed sswith 15 *y,v,sc,sev; lethal/CyO* females to isolate mutant animals. Both *nanos-Cas9* and *adp* sgRNA constructs are tagged with *y*^*+*^,*v*^*+*^. F2 individuals were selected for *y*^−^ *and v*^–^ to ensure the removal of the Cas9 and sgRNA sequences and balance *adp* mutations. Once F3 larvae appeared, the founder F2 individual was removed from the tube for sequencing. Ten mutant stocks were established using this method. All work in the rest of this paper was performed with the *adp*^*1*^ mutant. The *adp*^*1*^ mutant was backcrossed to *w*^*1118*^ for three generations to remove any extraneous mutations that may have occurred during mutagenesis. Backcrossing also allows for *w*^*1118*^ to be used as a control. Both the *adp*^*1*^ mutant and the *adp* duplication line were crossed into the same balancer line before being double balanced for rescue.

### Sequencing and primers

Founder F2 adults were squished with fresh squishing buffer (10 mM Tris pH 8.2, 25 mM NaCl, 1 mM EDTA, and 200 µg/mL Proteinase K). The lysate was incubated for 30 minutes at 37°C degrees and then for 10 minutes at 85°C. 2 µL of the lysate was used for sequencing. The following primers were used for PCR: 5’-AACAAGTGTCATAATCCTATCCACAGCA-3’ and 5’-TGCATGCAGCCAATATAGATCAAGATG-3’. PCR products were purified and sequenced using the same primers. Sequencing of *adp*^*1*^ showed a single insertion c.237_238insT resulting in an early stop codon p.Asp134Ter.

### Buoyancy assay

To indirectly test the fat content of *adp*^*1*^, we performed a buoyancy assay [29]. Approximately 20–40 late third instar larvae were collected from either *adp*^*1*^ or *w*^*1118*^ vials and placed in 50 mL conical tubes with a starting solution of 11.5 mL PBS and 9 mL 20% w/v Sucrose in PBS. Samples were swirled and inverted 2–3 times and settled for 2 min. The number of larvae floating was counted. 1 mL 20% sucrose was added to the conical, and samples were swirled, inverted, and settled. The number of floating larvae was recorded after each 1 mL addition. Additional sucrose was added, and floating larvae were counted until all larvae floated in each genotype. This experiment was repeated with 5 additional cohorts. The average sucrose concentration for all larvae to float in each genotype was compared using a paired t-test in Prism.

### Nile red staining

Late third-instar larval brains from *adp*^*1*^ and *w*^*1118*^ were dissected in PBS and then fixed with 4% paraformaldehyde in PBS for 30 min [30]. The brains were washed with PBST three times for 20 min each before incubating overnight at 4°C in 1 ug/mL Nile Red in PBST. Finally, brains were washed twice with PBST for 30 min each before being mounted for imaging.

### smFISH

#### Probes

We utilized the free ProbeDealer code for MATLAB to generate smFISH probes for *adp* [39]. The code, instructions, and *Drosophila* database are at https://campuspress.yale.edu/wanglab/ProbeDealer/. The *adp* input sequence was the whole mRNA transcript FASTA file from FlyBase. We selected to make 36 ‘sequential RNA fish’ probes outputted as ‘primary probe sequences’. The code automatically puts a 20-nucleotide secondary probe binding sequence on the 5’ and 3’ ends of each probe, making the final product 70 nucleotides along. We chose to remove the 20-nucleotide sequence from the 3’ end. The probes were ordered from Integrated DNA Technologies (IDT) with the “ssDNA oligo plate, 25 nmole scale, standard desalting” option. A single secondary probe was ordered also from IDT with the “ssDNA oligo, 250 nmole scale, HPLC purification” option using the 20-nucleotide secondary sequence and attaching Alexa Fluor 594 tag to the 3’ end.

Oligos were resuspended in RNase free water to 100 µM, and 50 µL each probe was combined into a single solution with total probe concentration as 100 µM.

All solutions were treated with DEPC or filtered to remove potential RNases and kept RNase free throughout the protocol. Vessels were treated with RNase ZAP and rinsed with RNase free solutions. Third-instar larval brains were dissected in PBS, fixed in 4% paraformaldehyde in PBS + 0.3% TritonX for 20 min. Samples were rinsed with PBS + 0.3% Tween 20 three times, washed for 15 min at room temp with PBS + 0.3% Tween 20 three times, then incubated in wash buffer (10% deionized formamide in 2X SSC) for 5 min at 37°C. Primary and secondary probes were diluted 1:250 in hybridization buffer (10% deionized formamide and 10% dextran sulphate in 2X SSC), incubated with tissue overnight at 37°C with gentle shaking on a Thermoshaker in the dark. Samples were rinsed three times in wash buffer, washed three times for 15 min in wash buffer at room temperature, and rinsed with PBS + 0.03 Tween 20 +DAPI. Brains were mounted in Slowfade Gold.

### Confocal microscopy

All the imaging was performed on a Zeiss LSM 710 confocal microscope with the 40X water immersion lens. A single brain lobe was centred in frame [20,21]. Zoom was set to 0.7, and scanning was done at speed 9. Z-stacks were set to encompass the entire z-range of the lobe, and the stack size was set at 2 µm.

#### Nile Red

The Alexa-fluor 488 channel was used for Nile Red imaging [40]. The frame size was 1024 × 1024 with a line averaging of 2.

#### smFISH

Images were optimized for the secondary probe (Alexa Fluor 594). A LD C-Apochromat 40×/1.1 W Korr M27 lens was used. All images were taken on the same day with the same laser power (5), gain (959), pinhole (1 airy unit), and scanning parameters (frame: 1024 × 1024, line average: 16, scan speed: 6). Post imaging processing changed the signal max from 255 to 115 in Imaris on all images.

#### Volume

Z stacks were set using Deadpan signal (647 channel) for volumetric analysis. The frame size was 512 × 512.

### Volume analysis

Analysis was performed using the IMARIS software with the surface function. To compute volume, we drew surfaces around every 5th z-stack, including the two farthest ends of the brain. The stacks were then compiled, and volume was generated automatically. The average volume was compared using a t-test in Prism.

### Nile red analysis

Analysis was performed using the IMARIS software with the surface and spots functions. First, all images were set to the same brightness and contrast settings (Minimum of 35.46, maximum of 255, and gamma or 2.12). Volume was computed under the surface tab as described above, then a mask of the surface was generated to be used as a region of interest in spots. Under the spots tab, we used the automatic spots counter with an estimated XY diameter of 1.96523 units, and the quality filter was set to 20%, allowing for the most lipid droplets to be counted without generating false positives. We then looked through the brain to remove false positives and add in missed droplets, therefore manually ensuring all lipid droplets were accounted for without false spots being counted. To count the large lipid droplets, we manually counted all droplets that were greater than 5 μm in diameter. For each brain, the number of lipid droplets was divided by the volume to compute the number of droplets/µm^3^. The average number of lipid droplets/µm [3] was then compared between groups with a t-test in Prism.

### Statistical analysis

All statistical analyses were performed using GraphPad Prism software. Independent t-tests for brain volume of neural stem cell knockdown, post-mitotic neuronal knockdown, *Oregon-R* vs *adp*^*60*^, lipid droplet analysis, and *w*^*1118*^ vs *adp*^*1*^ were done by selecting ‘t-tests’ under column analyses and ‘Unpaired’ under Experimental Design and assuming Gaussian distribution and equal SD. The paired t-test for WDTC1 rescue of buoyancy was performed by selecting ‘t-tests’ under column analysis and ‘Paired’ under Experimental Design and assuming Gaussian distribution and equal SD. Finally, the repeated measures one-way ANOVA for the buoyancy in Figure 2c was performed by selecting ‘One-Way ANOVA’ under Column Analyses, ‘each row represents matched, or repeated measures, data’ under Experimental Design, assume Gaussian distribution, and not assuming sphericity therefore using Geisser-Greenhouse correction. Under the ‘Multiple Comparisons’ tab, ‘Compare the mean of each column to the mean of every other column’ to allow for identification of rescue phenotypes. The p-values reported in figure legends were the *p* value under the Repeated Measures ANOVA Summary and the adjusted *p* values from the multiple comparisons.

## Supplementary Material

Supplemental Material

## Data Availability

Strains and plasmids are available upon request. The authors affirm that all data necessary for confirming the conclusions of the article are present within the article, figures, and tables.
