# Supplementary figures and images for: A novel adipose loss-of-function mutant in Drosophila
Source: Fly (Austin). 2024 May 13;18(1):2352938. doi: 10.1080/19336934.2024.2352938 (PMC11095658; doi:10.1080/19336934.2024.2352938)

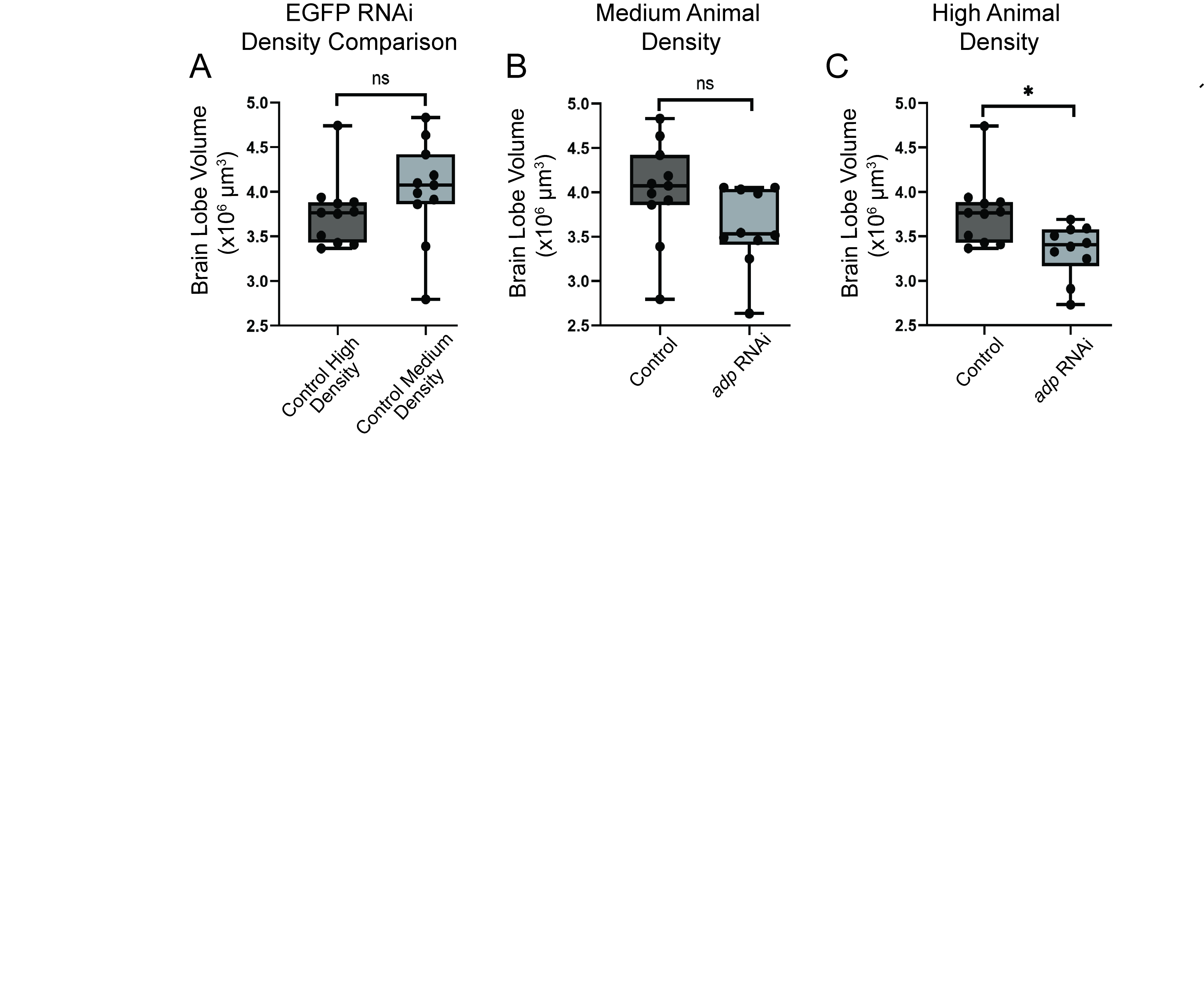

Supplement: Supplemental Material [file KFLY_A_2352938_SM3785.zip › Supplemental Figure 1 (1).tif]
